# Supplementary material for: Cell wall inhibitors increase the accumulation of rifampicin in Mycobacterium tuberculosis
Source: Access Microbiol. 2019 Mar 20;1(1):e000006. doi: 10.1099/acmi.0.000006 (PMC7470358; doi:10.1099/acmi.0.000006)
Supplement: Supplementary material 1 [file acmi-1-006-s001.pdf]

Materials. All chemicals used were purchased pure, from commercially available sources such as Sigma Aldrich, VWR, Fisher or other chemical vendors. ^{1}H NMR (300MHz) spectra were recorded on a Bruker Biospin NMR spectrometer. Thin layer chromatography was performed using Whatman silica gel 60 Å plates with florescent indicator and visualized using a UV lamp (254 nm). Flash chromatography was performed on Grace with GraceResolv Normal Phase disposable silica columns. Liquid chromatography electrospray ionization mass spectroscopy (LC-MS/ESI-MS) were acquired on an Agilent LC/MSD-SL with an 1100 HPLC and G1956B mass spectrometer with a Phenomenex Gemini 5 μm C18 110Å 50x3 mm column.

5-(4-(tert-butoxycarbonylamino)piperazine-1-carbothioamido)-2-(6-hydroxy-3-oxo-3H-xanthen-9-yl)benzoic acid (3): To a solution of fluorescein isothiocyanate isomer 1 (389 mg, 1 mmol) and tert-butyl piperazin-1-ylcarbamate (201 mg, 1 mmol) in dimethylformamide (5 mL) was added triethylamine (200 mg, 2 mmol). The reaction mixture was stirred at room temperature for 3 h. Saturated sodium chloride solution and ethyl acetate was added to the reaction mixture. The aqueous layer was extracted with ethyl acetate. The combined organic layer was dried over anhydrous sodium sulfate and concentrated on rotary evaporator. The crude product was purified by flash column chromatography on silica (hexanes:ethyl acetate 1:0 to 0:1) to yield **1** as a red solid (0.348 mg, 59 %).

5-(4-aminopiperazine-1-carbothioamido)-2-(6-hydroxy-3-oxo-3H-xanthen-9-yl)benzoic acid (4): To **1** (200 mg, 0.33 mmol) was added 4 N hydrochloric acid in methanol (5 mL). The reaction mixture was stirred at room temperature for 36 h. The reaction mixture was concentrated and then ethyl acetate was added to precipitate the product as the hydrochloride salt which was neutralized with trimethylamine and carried forward without further purification (145 mg, 90%).

RIF-FITC: 3-Formyl rifamycin sv (145 mg, 0.02 mmol) and 5-(4-aminopiperazine-1-carbothioamido)-2-(6-hydroxy-3-oxo-3H-xanthen-9-yl)benzoic acid (100 mg, 0.2 mmol) were dissolved in methanol (4 mL) and the reaction was stirred at room temperature for 36 h. The reaction mixture was diluted with ethyl acetate and the formed precipitate was collected by filtration. The precipitate was purified by flash chromatography on silica gel (dichloromethane: methanol 1:0 to 90:10) to yield the desired product as an orange solid (55 mg, 23%). The purity of the final product was >95% as determined by HPLC analysis conducted on an Agilent 1100 HPLC/MS system (Phenomenex Gemini C18 column, 5 μm, 3 x 50 mm, 0.45 mL/min, UV 254

nm, room temperature) with gradient elution (5-95% acetonitrile in water over 8 min with all solvents containing 0.05% formic acid.
